# Perineal Incision for the Surgical Management of Extremely Proximal Internal Penile Fractures—A Case Series and Review of Literature

**DOI:** 10.1155/criu/7921626

**Published:** 2025-04-14

**Authors:** Anas Khan, Hester Lacey, James Brittain, Charles Coker, Ruairidh Crawford

**Affiliations:** ^1^Department of Urology, University Hospitals Sussex NHS Foundation Trust, Princess Royal Hospital, Haywards Heath, UK; ^2^Brighton and Sussex Medical School, University of Sussex, Brighton, UK

**Keywords:** MRI, penile fracture, perineal approach

## Abstract

This case series presents four cases of extreme proximal internal corpus cavernosum (EPICC) penile fractures. Patients, aged 33–53, presented with penile trauma primarily during sexual intercourse, exhibiting atypical symptoms sparing the penis but with significant perineal and scrotal bruising. Diagnosis was confirmed via penile magnetic resonance imaging (MRI), revealing fractures at the penile base. All patients underwent surgical repair through a midline perineal incision, with no long-term complications reported. This series highlights the value of MRI for diagnosing atypical fractures and supports a perineal surgical approach for optimal exposure and repair in EPICC fractures.

**Summary**

• Fractures of the base of the penis should be suspected where there are normal penile examination findings, but clinical suspicion remains high.
• Prompt penile MRI can provide accurate location for internal penile fractures.
• Perineal incision rather than circumferential degloving approach should be adopted for extreme proximal internal penile fractures.

## 1. Introduction

Penile fractures are a rare urological emergency with an estimated incidence of 1 per 100,000 male population, with extreme proximal internal corpus cavernosum (EPICC) penile fractures being an even rarer entity [1, 2]. These injuries occur just below the ischiopubic rami at the crus of the cavernosal bodies. In this area, the anchoring connective tissues offer additional support and strength to the corpora cavernosa making injury difficult [2]. However, the surrounding tunica albuginea fascia decreases to 0.25-mm thickness when erect making it susceptible to traumatic injury [3]. The most common cause is trauma during sexual intercourse in which the erect penis hits the pubis or perineum of the partner leading to rupture in the tunica surrounding the corpora cavernosa. Timely surgical intervention is crucial to restore both functional and structural integrity and reduce the risk of long-term complications such as erectile dysfunction and penile curvature.

## 2. Case Presentation

This case series describes four cases of EPICC presenting to the authors' institution between December 2022 and June 2024. The patients (Patients A, B, C, and D) presented with varying degrees of penile trauma, associated with sexual intercourse or direct trauma. Patient age ranged from 33 to 53 years, with the common presenting symptoms of bruising, swelling, and immediate detumescence following traumatic insult.

**Figure 1:**
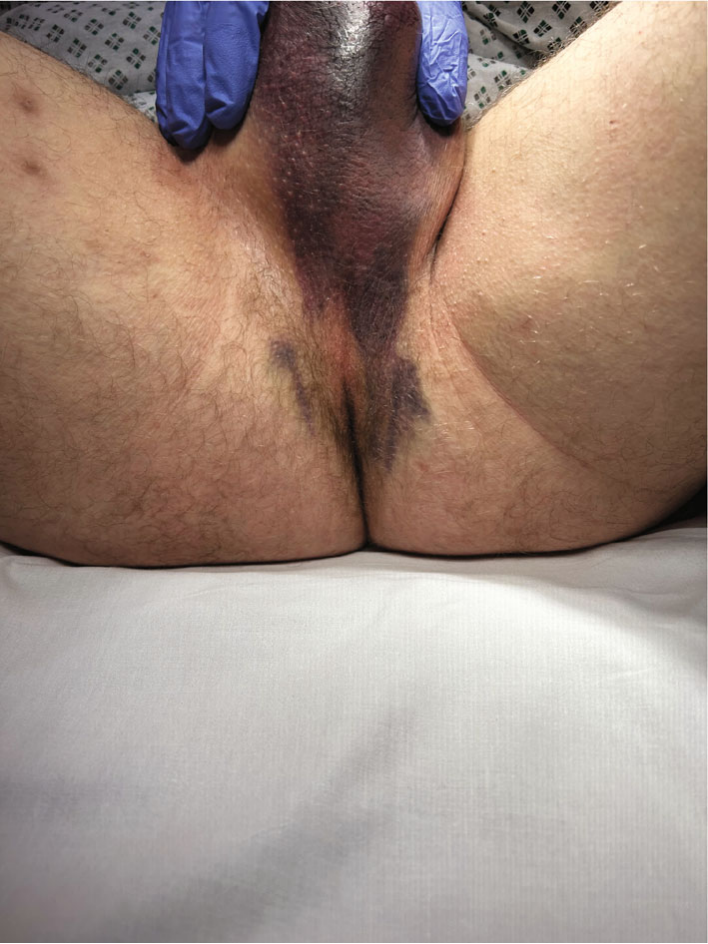
Perineal and scrotal bruising pattern seen in Patient B.

Patient A was a 53-year-old male who experienced penile trauma during sexual intercourse when his penis slipped out and was bent against his partner's perineum. He reported a “pop” sound, immediate detumescence, and significant leftward angulation of the penis, accompanied by blood from the urethral meatus and bruising at the base of the penis and scrotum. Patient B, a 36-year-old male, also sustained his injury during sexual intercourse during the previous night. He described feeling a twinge of pain that radiated into the scrotum, followed by severe pain, nausea, and light-headedness. On examination, there was swelling of the proximal penile shaft and a bruising pattern extending from the midpenile shaft down into the scrotum and perineum (Figure 1). The distal penis appeared spared.

Patient C was a 33-year-old male, who presented after trauma involving his erect penis being forcibly pushed down during micturition, leading to bruising of the proximal shaft and scrotum. He sought medical attention on the same day of the injury, reporting pain and visible bruising. Finally, Patient D was a 45-year-old male, who sustained his penile injury during sexual intercourse in the “missionary” position. He heard a “pop,” followed by immediate detumescence and swelling. On examination, there was generalized bruising of the perinium and scrotum but no obvious defect or angulation of the penis. He had minimal tenderness on palpation.

In terms of medical history, the cohort of patient lacked major significant comorbidities. Patient A had a history of anxiety, depression, and ocular hypertension, while Patient C had benign intracranial hypertension. Patients B and D were otherwise fit and well.

**Figure 2:**
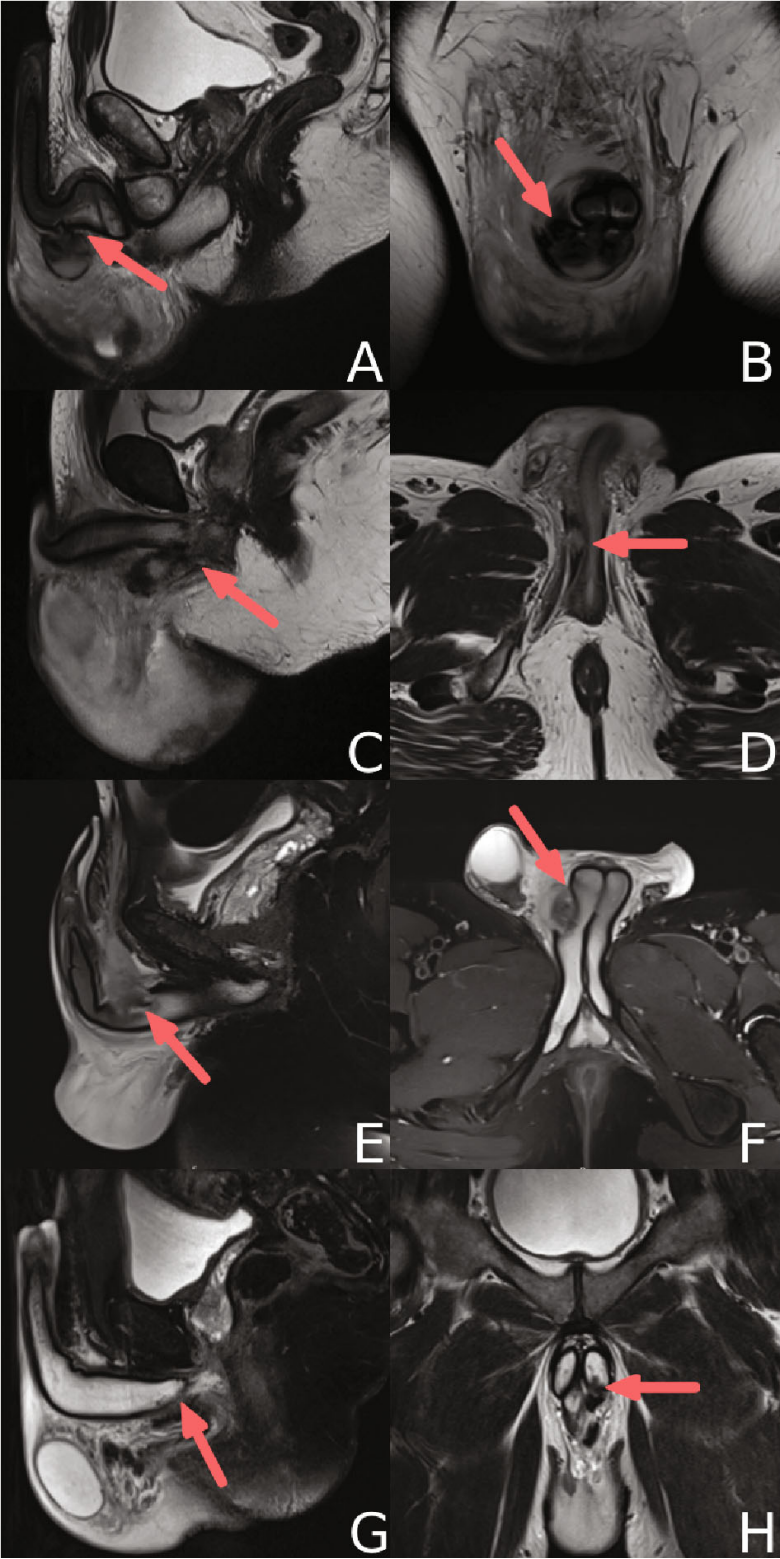
Penile MRI slices from admission. Red arrow indicates location of penile fracture. Patient A: T1-weighted MRI sagittal (A) and coronal (B). Patient B: T1-weighted MRI sagittal (C) and axial (D). Patient C: T2-weighted MRI sagittal (E) and axial (F). Patient D: T2-weighted MRI sagittal (G) and coronal (H).

All patients had routine laboratory blood tests on admission which were mostly within normal values. The exception were Patients A and B who had slightly elevated inflammatory markers on admission with white cell counts (WCCs) of 15.4 and 11.3, respectively. Examination findings were atypical for penile fracture (penises were soft with no palpable hematoma and only mild bruising in one case); hence, all patients underwent penile magnetic resonance imaging (MRI) testing within 24 h, revealing proximal penile fractures (Figure 2). Ultrasound was not performed.

All four patients were admitted to hospital and prescribed analgesia, antiemetics, and low molecular weight heparin prophylaxis. Three patients were also started on intravenous co-amoxiclav antibiotics. Patients were kept nil by mouth while awaiting cross-sectional imaging. Once a penile fracture was identified, patients where prepared for surgical repair in the operating theatre.

**Figure 3:**
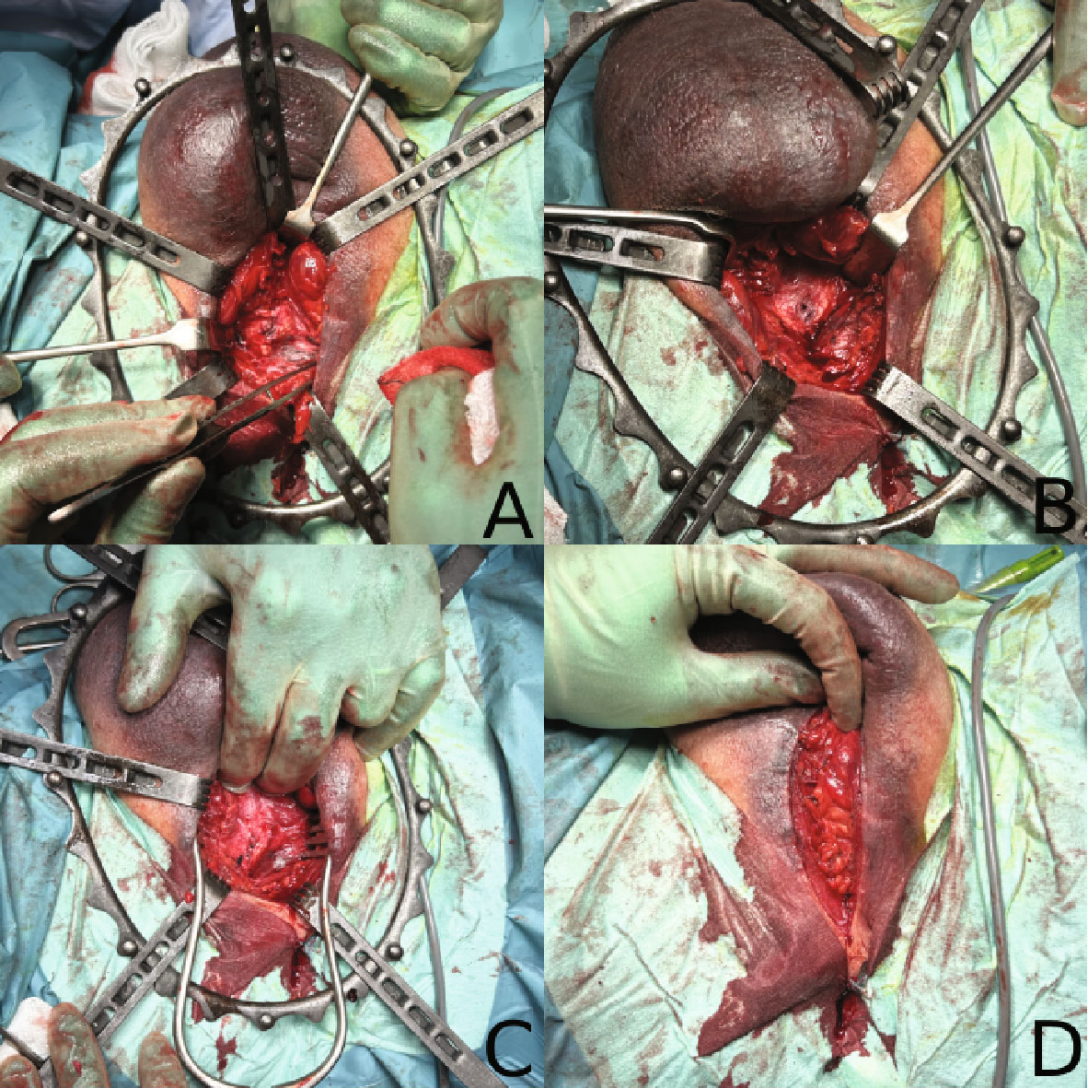
Intraoperative images from Patient B showing midline perineal approach (A), tunical defect in the right ventral root of the corpus spongiosum (B), closure of defect with interrupted 2-0 polydioxanone sutures (C), and closure in layers (D).

Surgical repair was performed under general anesthetic in lithotomy position. Three patients underwent surgical repair within 24 h of presentation with Patient C having his repair delayed by 72 h due to lack of uroradiologists. Approach for all patients was the same involving a vertical midline incision in the perineum, with dissection down to the corporal bodies adjacent to the bulbar urethra (Figure 3). A Turner-Warwick retractor was used to maintain exposure. Here, the hematoma was evacuated and the defect in the tunica albuginea closed with interrupted polydioxanone sutures. A partial urethral injury was also treated in Patient A with a dorsal urethrotomy and closure with polyglactin 910 sutures over a Foley catheter. Closure was performed in layers with polyglactin 910 to eliminate dead space and skin closed vertical mattress sutures. Total operating time ranged between 56 and 73 min, with minimal blood loss.

Postoperatively, the patients were discharged the same day of procedure, with advice to avoid strenuous activity and sexual intercourse for at least 6 weeks. Due to the urethral repair, Patient A was also sent home with a urinary catheter. Patient B developed a wound infection 2 weeks following discharge that was effectively treated with oral antibiotics. No other significant postoperative complications were reported.

Patients were seen in clinic between 3 and 6 weeks after discharge. Patient A also had a pericatheter urethrogram 3 weeks postsurgery to ensure no leak in the urethra before removal of catheter. All patients made good progress, with return of erections. At 3 weeks, only Patient B still had some bruising at the base of the penis which later resolved.

## 3. Discussion

Both British Association of Urological Surgeons (BAUS) and European Association of Urology (EAU) guidelines recommend surgical exploration within 24 h of presentation; however, delays of up to a week or longer in the case of urethral injury should not prevent the decision to operate [3, 4]. Both guidelines preferred surgical approach for penile fractures, especially in cases where the site of defect is unknown, is the subcoronal circumferential degloving incision, providing wide exposure of the penile shaft and access to repair both corporal bodies and any concomitant urethral injury. However, both societies recognize the use of a penoscrotal incision over the site of injury if possible. Regarding penile fractures at the root of the penis, there are no recommendations or guidelines for optimum management or surgical approach. In cases of EPICC fractures, degloving the penis may not allow adequate exposure of the corporal injury. In this case series, a more tailored approach involving midline perineal incisions has been successfully demonstrated.

A systematic literature review for other reports of EPICC fractures has revealed only 10 cases, although it is challenging to say if these are all true EPICC injuries at the root of the penis as MRI images were not provided by all authors (Supporting Information). Age ranged from 21 to 47 years old, and surgical intervention was performed between 12 h and 8 days after presentation. The majority were explored via a midline perineal incision similar to the presented cases [5–11]. 3-0 polydioxanone was most commonly used to repair the tunica defect, in line with BAUS and EAU guidelines with only Pruthi et al. using 2-0 polyglactin. These patients made good recovery with no significant complications at follow up. Blondaeu at al. also described the first two cases of conservatively managed very proximal penile fractures in a 25- and 38-year-old. Swelling, bruising, and pain had resolved by 6 weeks of follow-up with no new reported complications. It is clear that a perineal approach allows for targeted exploration and minimizes complications such as edema, reduced sensation, and poor cosmesis.

Classical distal or midshaft penile fractures present with a popping noise, immediate detumescence, and a typical “eggplant deformity” of the penis. In this case series, four patients presented with extremely proximal penile fractures resulting from sexual intercourse or accidental trauma. Here, a common presenting symptom was scrotal hematoma and perineal bruising. The distal penis was spared of bruising in three patients (Patients A, B, and C) and the whole penis examined normally in one patient (Patient D). Looking at the literature, 80% of patients with EPICC fractures had normal penile examination on presentation with no swelling or bruising [2, 5, 7–12]. Instead, scrotal hematoma (80%) and perineal bruising (80%) were common in this cohort; particularly, a “butterfly” pattern of ecchymosis across the perineal and upper thigh region was described in over half of the cases in the literature. These atypical symptoms found in cases of EPICC fractures pose diagnostic challenge, further emphasizing the importance of MRI in suspected cases. This is highlighted by Rezaee at al. who reported a case of missed proximal penile fracture who underwent a traditional degloving exploration without cross-sectional imaging. The authors failed to identify any corporal injury at the time of surgery. The patient developed worsening pain the following day, and a penile and pelvic MRI performed showed an internal penile left corpus defect. He was taken back to theatres for repair via a perineal incision at 8 days post injury. At follow-up, he reported a new left curvature in the penis [2].

For suspected classical shaft penile fractures, preoperative ultrasound could allow surgical planning and incision over the tunica defect. However, if there is clinical or ultrasound uncertainty or EPICC is suspected on examination, then an MRI should be performed which could save an unnecessary devolving, but we would struggle to make this recommendation given the variability of MRI access nationally. MRI's enhanced ability to delineate soft tissue injuries with high-contrast resolution was particularly valuable in our cases, especially when the physical presentation was ambiguous. One must bear in mind the associated costs of these investigations with MRI often being quoted as twice as expensive as ultrasound [13]. Additionally, this institutions experience is that penile ultrasounds and MRIs can usually only be performed and reported by uroradiologists, which appears to align with the Royal College of Radiology guidelines suggesting that these are specialist investigations, which further impacts the ability to standardize these investigations for working up potential penile fractures [14].

The most significant long-term concern following a penile fracture is the potential for erectile dysfunction, penile curvature, or painful erections due to the formation of fibrotic tissue. In these presented cases, all patients reported satisfactory postoperative erectile function, with no significant curvature or deformity.

## 4. Conclusion

Penile fracture, particularly of EPICC fracture, remains a rare but critical condition requiring prompt diagnosis and surgical intervention. This case series emphasizes the value of MRI cross-sectional imaging, especially for atypical presentations where clinical suspicion remains high. A midline perineal approach also appears appropriate for repairing the tunica albuginea at the penile base, with direct access to the corporal defect, which a degloving incision would make almost impossible. The absence of long-term complications suggests that the timely surgical intervention was effective and that perineal approach should be adopted for this cohort of patients. The benefit of imaging may be worth a small-time delay, and further evidence is needed before recommendations are made.

## Supporting Information

Supporting Information Additional supporting information can be found online in the Supporting Information section. Page S1: Search strategy for identifying cases of proximal penile fracture in existing literature and flowchart showing how many papers were included. Page S2: Table of proximal penile fracture case reports in literature.
