# Supplementary material for: Perineal Incision for the Surgical Management of Extremely Proximal Internal Penile Fractures—A Case Series and Review of Literature
Source: Case Rep Urol. 2025 Apr 14;2025:7921626. doi: 10.1155/criu/7921626 (PMC12011461; doi:10.1155/criu/7921626)
Supplement: Supporting Information — Additional supporting information can be found online in the Supporting Information section. Page S1: Search strategy for identifying cases of proximal penile fracture in existing literature and flowchart showing how many papers were included. Page S2: Table of proximal penile fracture case reports in literature. [file 7921626.f1.docx]

# Supplementary files

MEDLINE search strategy: (proximal OR internal OR root OR base OR crura OR crus) AND (penile OR penis OR corpora* OR caversosum OR cavernosa OR corpus) AND (fracture* OR tear* OR disruption OR injur*)


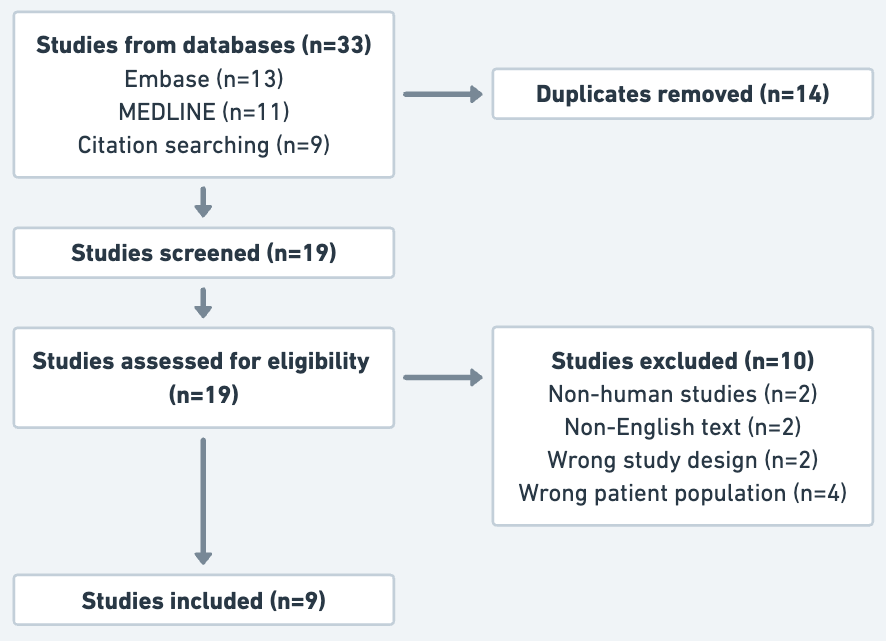


| **Study** | **Year** | **Age** | **Cause** | **Butterfly pattern** | **Perineal bruising** | **Scrotal bruising** | **Normal penis** | **Investigations** | **Injury location** | **Management** | **Complications** |
| --- | --- | --- | --- | --- | --- | --- | --- | --- | --- | --- | --- |
| Darves-Bornoz et al | 2013 | 47 | Sexual intercourse | No | Yes | Yes | Yes | MSU, US, MRI | tear of the tunica overlying theright proximal corpus cavernosum | Surgery - midline perineal | Not reportted |
| Rezaee and Gross | 2021 | 32 | Sexual intercourse | No | No | Yes | Yes | None initially then US, MRI day 1 post operation | internal penis left corpus | Surgery - degloving and exploration | Worse pain day 1 post op. New curvature to left at follow up |
| Kyriazis et al | 2022 | 25 | Sexual intercourse | No | Yes | Yes | Yes | MSU, MRI | defect within the tunica over the ventral aspect of the right corpus cavernosum at the penile root | Surgery - midline perineal | Nil |
| Buckland et al | 2024 | 30s | Sexual intercourse | No | No | No | Yes | None initially then US & MRI 1 week later | proximal right corpus cavernosum defect | Surgery - midline perineal | Nil |
| Pruthi et al | 2000 | 21 | Sexual intercourse | Yes | Yes | No | Yes | Cavernosography, retrograde urethrogram | left proximal corporeal body 1.5cm from pubic insertion | Surgery - midline perineal | Nil |
| Harada et al | 2021 | 25 | Rolling over in bed | Yes | Yes | Yes | No | US, MRI | tear to the ventral tunica of the left crus penis near the bulbospongiosus muscle | Surgery - midline perineal | Nil |
| Gochenaur et al | 2022 | 33 | Sexual intercourse | Yes | Yes | Yes | Yes | MSU, US, MRI | tunica overlying the crura of the right corpus cavernosum | Surgery - midline perineal | Nil |
| Hina et al | 2022 | 39 | Unknown | Yes | Yes | Yes | Yes | MRI | base of the right corpora cavernosa | Surgery - midline perineal | Nil |
| Blondeau et al | 2023 | 25 | Sexual intercourse | Yes | Yes | Yes | Distal | US, MRI | right proximal corpus cavernosum | Conservative | Nil |
| Blondeau et al | 2023 | 38 | Sexual intercourse | Yes | Yes | Yes | Yes | US, MRI | tear of the medial aspect of the left corpora adjacent to the penile bulb | Conservative | Swelling/bruising at 3 weeks with sex, resolved eventually |

Table of proximal penile fracture case reports in literature
